# Modulated Expression and Activities of *Ruditapes philippinarum* Enzymes After Oxidative Stress Induced by Aerial Exposure and Reimmersion

**DOI:** 10.3389/fphys.2020.00500

**Published:** 2020-05-25

**Authors:** Hongtao Nie, Kunyin Jiang, Zihan Zhou, Baiying Guo, Dongdong Li, Xiwu Yan

**Affiliations:** Engineering and Technology Research Center of Shellfish Breeding in Liaoning Province, College of Fisheries and Life Science, Dalian Ocean University, Dalian, China

**Keywords:** *Ruditapes philippinarum*, aerial exposure, reoxygenation, oxidative stress, enzymatic activity, gene expression

## Abstract

*Ruditapes philippinarum*, is an economically and scientifically important bivalve mollusk. Its tolerance of aerial exposure has long been considered an important trait for its survival under acute environmental stress. In this study, the effects of air exposure at high and low temperatures (28 and 4°C) on the survival, antioxidant and metabolic enzyme activities, and the expression of antioxidant and immune-related genes in *R. philippinarum* were investigated. The activities of antioxidant and metabolic enzymes [superoxide dismutase (SOD), α-amylase, and proline hydroxylase (PHD)] were significantly affected by aerial exposure and reimmersion (reoxygenation) at both low (4°C) and high (28°C) temperatures. Moreover, the mRNA expression of α-amylase, SOD, and C-type lectin was also examined, which reveals these genes were significantly affected by aerial exposure challenge. In addition, the effects of aerial exposure and reimmersion on survival rate were calculated to evaluate the recovery capacity of Manila clam after aerial exposure at high and low temperatures. All individuals survived under low temperature aerial exposure for 24 h and reimmersion for 120 h. However, individuals died after reimmersion for 12 h following high temperature aerial exposure, and mortality peak occurred at 48 h. These data indicate that long-term aerial exposure during the transportation of clams should be in a low temperature environment. This study demonstrates that enzyme expression and activities linked to the stress response increase during the aerial exposure of *R. philippinarum* and provide useful information for future work on the molecular basis of tolerance of aerial exposure stress.

## Introduction

The Manila clam, *Ruditapes philippinarum*, is an economically and scientifically important marine bivalve species with a wide geographic distribution, extending from Europe to Asia (Zhang and Yan, 2006, 2010). Manila clam has a great capacity to adapt to new environments (Solidoro et al., 2000). Its ability to cope with abiotic and biotic stresses is vital to its survival because it has an intertidal benthic lifestyle, and is therefore subjected to cycles of emersion and reimmersion during the tidal cycle. Because juvenile clams settle in muddy or sandy sediments in the intertidal zone and live buried several centimeters deep (Yin et al., 2017), they experience daily rhythms of air exposure throughout their lives, which impose physiological stresses, including water loss, oxygen deficiency, thermal stress, and food limitation (McMahon, 1988; Byrne et al., 1990; Hiong, 2004; Nie et al., 2016, 2017).

A tolerance of aerial exposure has long been considered an important trait for survival under acute environmental stress, especially for aquatic animals (Li et al., 2017). Aquatic animals in intertidal areas must cope with being out of the water at regular intervals. Emersion times are longest for animals in the high intertidal zone, so these individuals are often subjected to aerial exposure. Many aquatic species, such as shellfish, can also experience aerial exposure during their harvest and transportation. Therefore, the tolerance of aerial exposure in shellfish is an interesting ecological phenomenon, the molecular and physiological mechanisms of which require clarification, and have received considerable attention in recent years (Romero et al., 2007; Li et al., 2017). Previous studies have reported several effects of aerial exposure on the molecular, physiological, and biochemical responses (metabolic costs, antioxidant defenses, fermentative metabolism, etc.) of aquatic mollusks. Oxygen sensors and several pathways (oxidative stress, heat shock stress, energy metabolism, immune functions, etc.) have been implicated in the adaptive response to changes in oxygen availability in several bivalves, including mussels (Widdows and Shick, 1985; Almeida et al., 2005; Almeida and Bainy, 2006; Kawabe and Yokoyama, 2012; Woo et al., 2011; Giannetto et al., 2015, 2017; Nogueira et al., 2017), oysters (Sussarellu et al., 2010; Piontkivska et al., 2011), and others (Widdows et al., 1979; Philipp et al., 2012). Bivalves are known to utilize anaerobic metabolic pathways when their tissues are deprived of oxygen (Zwaan, 1977). However, little is known about the mechanisms underlying the regulation of the physiological and immune responses to aerial exposure in the Manila clam (Yin et al., 2017).

Aerial exposure greatly affects the immune functions and stress resistance of shellfish, and can lead to death from oxidative damage, antioxidation, and immunosuppression (Malagoli et al., 2007; Li et al., 2017). α-amylase is a digestive and metabolic enzyme associated with growth and metabolism. α-amylase hydrolyzes amylose to glucose during digestion, which promotes the growth and development of organisms (Sellos et al., 2003). It is the key enzyme in carbohydrate assimilation in mollusks (Qian et al., 2003). Proline hydroxylase (PHD) is a metabolic enzyme that allows the body to adapt to hypoxic environments (Piontkivska et al., 2011), and regulates the stability of hypoxia inducible factor (HIF) in oxygen utilization. The inhibition of PHD during oxygen limitation stabilizes HIF and permits cells to adapt to hypoxia (Nguyen and Durán, 2016). Superoxide dismutase (SOD) is an immune-related antioxidative enzyme, which eliminates the superoxide anions (free radicals) produced by immune reactions in the body (Afonso et al., 2007). In addition, C-type lectins (CTLs) are pattern recognition receptors (PRRs) that play important roles in the immune responses and defenses of shellfish (Li et al., 2019). The identification of the genetic mechanisms regulating these physiological, immunological, and biological processes will provide key insights into the adaptive responses of marine bivalves to aerial exposure and aerial exposure stress (Ekblom and Galindo, 2011; Place et al., 2012).

In this study, the changes in physiological indices during the process of resistance to air exposure were investigated by monitoring the changes in the metabolic and antioxidant enzyme activities of *R. philippinarum* under the stress imposed by aerial exposure and reimmersion in water at different temperatures. We also monitored the different mortality rates during these processes under different conditions. This study provides new insights into the physiological and biochemical mechanisms underlying aerial exposure tolerance in *R. philippinarum* and extends our understanding of the physiological changes that occur in response to low oxygen availability, the fundamental adaptive physiology of this organism, and the molecular mechanisms operating during aerial exposure.

## Materials and Methods

### Experimental Clams

The experimental clams were collected from Jinshitan, Dalian, China. Those clams had an average shell length of 35.05 ± 2.24 mm and an average weight of 8.96 ± 1.21 g. Before the experiment, all the Manila clams were acclimatized in aerated seawater (31 ± 1 ppt) at 14 ± 1°C for 1 week in lab condition (Li et al., 2017). All the clams were fed spirulina powder daily for 1 week and fasted for 2 days before aerial exposure, and the water was exchanged daily to remove the waste products from the marine invertebrates. Other water parameters were measured during the experiment (pH 8.2 ± 0.2; dissolved oxygen, 8.5 ± 1.0 mg/L). *R. philippinarum* is not an endangered or protected species, so there was no need for specific procedures or approval were required in this study.

### Challenge and Sampling

Totally 360 Manila clams were averagely divided into high temperature experimental group (TH), high temperature control group (CH), low temperature experimental group (TL), and low temperature control group (CL). Each group consisted of three replicates with a total number of 90 clams. The high-temperature experimental group was aerially exposed at 28°C, and the low-temperature experimental group at 4°C (Wang, 2010) for 3, 6, 12, or 24 h. The aerially exposed groups were then respectively restored to their original tanks at 4 or 28°C for 120 h. Gill and hepatopancreas samples were collected from three replicates of experimental groups and control groups at different time points (aerial exposure: 3, 6, 12, and 24 h; reimmersion: 3, 6, 12, 24, 48, 72, and 96 h).

For the measurement of enzyme activities, about 0.1 g samples of gill and hepatopancreas were pooled within the same group and a 0.9 volume of normal saline was added according to weight (g): volume (ml) = 1: 9. The rest of the collected gill and hepatopancreas samples were stored at −80°C in a freezer for subsequent RNA extraction.

The mortality rates of the Manila clams after aerial exposure and reimmersion in the experiment groups and the Manila clams in control groups were calculated at different times during the experiment. In this work, the shell of the clam opened and could not be closed by the adductor muscle, and then the clam was classified as dead.

### Measurement of Enzyme Activities

For the measurement of enzyme activities, gill and hepatopancreas samples (about 0.1 g) were respectively pooled within the same group and a 0.9 volume of normal saline was added according to weight (g): volume (ml) = 1: 9. The samples were ground at low temperature (in an ice box) with an electric tissue grinder, and centrifuged at 2500 rpm in a freezing centrifuge at 4°C for 10 min. An aliquot (200 μL) of the supernatant was diluted with 0.9% normal saline in a 1:4 ratio for testing. Each enzyme activity index and protein concentration was measured three times by SpectraMax i3 (Molecular Devices, CA, United States) in all replicates.

All samples were tested with α-amylase, SOD, and PHD analysis kits manufactured by Nanjing Jiancheng Bioengineering Institute (Nanjing, China) according to the protocols of the manufacturer.

### Total RNA Extraction

Total RNA was extracted with TRIzol Reagent (TRIzol^®^ Plus RNA Purification Kit, Invitrogen, Carlsbad, CA, United States), according to the manufacturer’s protocol. The total RNA concentration was measured with a NanoDrop 2000c UV/Vis spectrophotometer (Thermo Fisher Scientific, Madison, NY, United States). The quality of the total RNA was confifirmed by electrophoresis on 1% agarose gel (Supplementary Figure S1). The total RNA was extracted from each tissue of *R. philippinarum*, reverse transcribed to cDNA, and stored at −20°C.

### Tissue Distribution and mRNA Expression of α-Amylase, SOD, and RpCTL in Manila Clams Under Aerial Exposure Analyzed With Reverse Transcription (RT)-Quantitative PCR (qPCR)

For the tissue expression analysis, the mantle, gill, siphon, adductor muscle, foot, and hepatopancreas tissues were collected from three unchallenged clams, and the total RNA was extracted with TRIzol Reagent (Invitrogen). The first-strand cDNA was synthesized with the QuantiTect^®^ Reverse Transcription Kit (TaKaRa Bio, Shiga, Japan), according to the manufacturer’s instructions. For the expression analysis of α-amylase, SOD, and RpCTL mRNAs in the Manila clams under aerial exposure, the gills and hepatopancreases were collected from three challenged clams in the *R. philippinarum*.

The qPCR was used to determine the mRNA expression levels of α-amylase, SOD, and RpCTL, with β-actin as the internal control, with the SYBR Green Master kit (Roche, Basel, Switzerland), according to the manufacturer’s protocol. The primers for qPCR are listed in Supplementary Table S1. The experiments were performed in triplicate, with three biological replicates of each sample. qPCR was performed with the Real-Time Detection System (Roche 480 LightCycler), using the SYBR ExScript qRT Kit (TaKaRa), in a total volume of 20 μL that contained 10 μL of SYBR^®^ Premix Ex Taq II (2×), 0.8 μL of each primer, 2 μL of cDNA, and 6.4 μL of H_2_O. The thermal cycling protocol was 94°C for 5 min, and 40 cycles of 94°C for 30 s, 60°C for 30 s, and 72°C for 30 s. The expression of α-amylase, SOD, and RpCTL mRNAs was normalized to that of β-*actin* mRNA, and the quantitative differences in expression between the different samples were calculated with the 2^–ΔΔCT^ method (Livak and Schmittgen, 2001).

### Statistical Analysis

All groups were analyzed with one-way analysis of variance (ANOVA), followed by an unpaired two-tailed *t*-test. *P* < 0.05 was deemed to indicate statistically significant differences and *P* < 0.01 highly significant differences. All data are expressed as means ± standard errors (SE). One-way ANOVA followed by Duncan’s multiple comparison test was used to compare the effects of hypoxia on the three enzyme activities (α-amylase, SOD, and PHD). Differences were considered significant at *P* < 0.05.

## Results

### Effects of Aerial Exposure on α-Amylase Activity in Manila Clam

**FIGURE 1:**
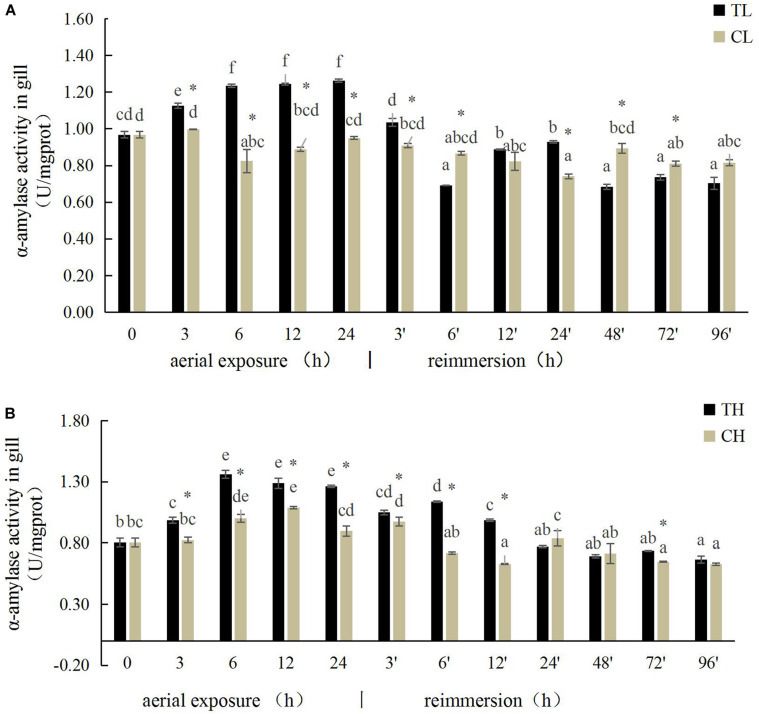
Effects of aerial exposure on α-amylase in gill of *Ruditapes philippinarum* under low temperature **(A)** and high temperature **(B)**. TL, low temperature experimental group; CL, low temperature control group; TH, high temperature experimental group; CH, high temperature control group. Asterisk represents significant difference in independent *t*-test in the control group at the same time; same lowercase letters are not significant, and different lowercase letters for difference. The same below.

The α-amylase activity in the gills of the Manila clam under aerial exposure at low temperature first increased significantly (3, 6, 12, and 24 h) and then decreased (3 and 6 h) after reimmersion (*P* < 0.05) (Figure 1A). A similar pattern in α-amylase activity was observed in the gills of the Manila clam under aerial exposure at high temperature (Figure 1B).

**FIGURE 2:**
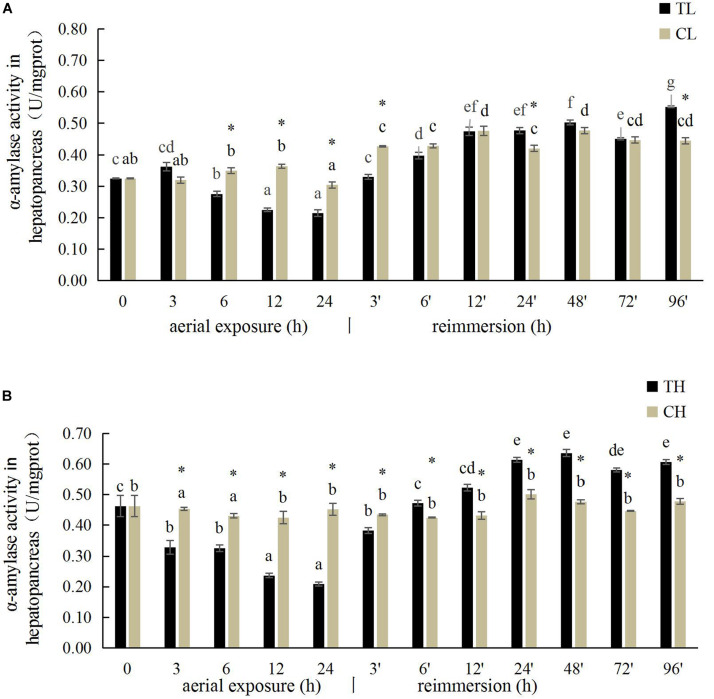
Effects of aerial exposure on α-amylase in hepatopancreas of *R. philippinarum* under low temperature **(A)** and high temperature **(B)**.

The hepatopancreatic α-amylase activity in the Manila clam under aerial exposure at low temperature is shown in Figure 2. With prolonged aerial exposure, the hepatopancreatic α-amylase activity first decreased significantly at 6, 12, and 24 h, and then increased significantly at 3 and 24 h after reimmersion (*P* < 0.05) (Figure 2). More significant variation in the hepatopancreatic α-amylase activity was observed in the Manila clam under aerial exposure at high temperature (Figure 2B).

### Effects of Aerial Exposure on SOD Activity in Manila Clam

**FIGURE 3:**
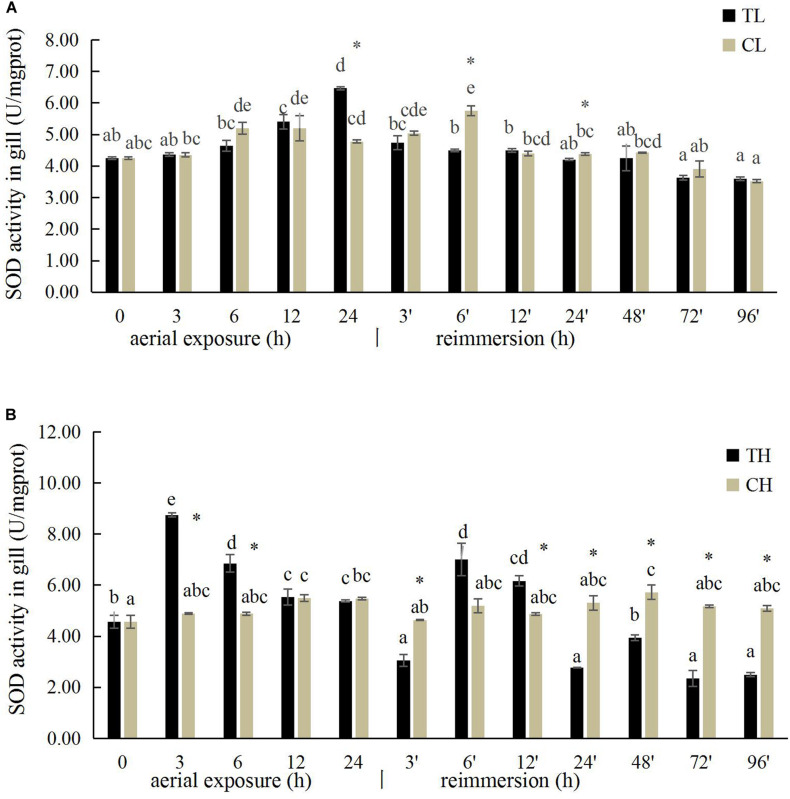
Effects of aerial exposure on superoxide dismutase (SOD) in gill of *R. philippinarum* under low temperature **(A)** and high temperature **(B)**.

**FIGURE 4:**
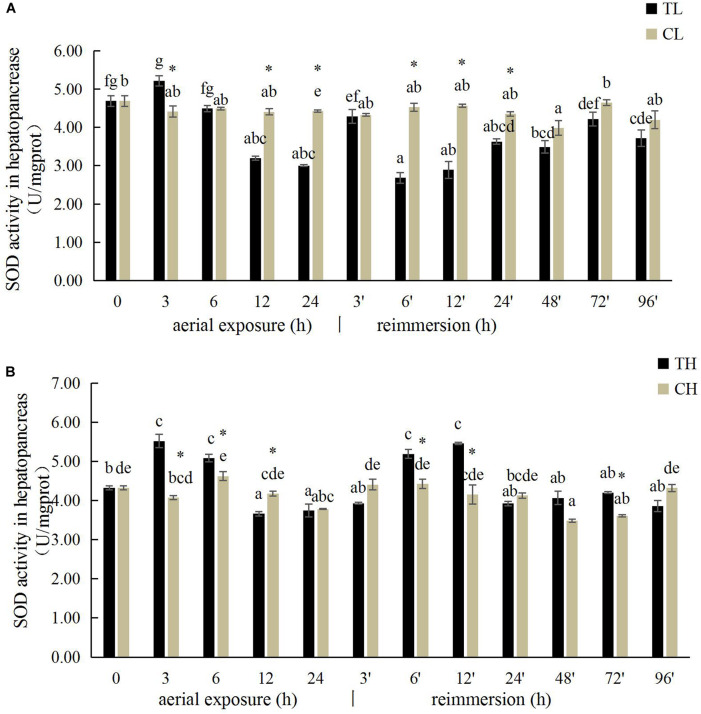
Effects of aerial exposure on superoxide dismutase (SOD) in hepatopancreas of *R. philippinarum* under low temperature **(A)** and high temperature **(B)**.

The SOD activity in the gills of *R. philippinarum* increased significantly after 24 h under aerial exposure stress at low temperature (*P* < 0.05), whereas a significant reduction in SOD activity was observed at 6 and 24 h in the gills of *R. philippinarum* after reimmersion (Figure 3A). At high temperature, the SOD activity in the gills of *R. philippinarum* increased significantly after 3 and 6 h under aerial exposure stress, whereas it was significantly reduced at 3, 24, 48, 72, and 96 h after reimmersion (*P* < 0.05) (Figure 3B). Under aerial exposure at low temperature, the hepatopancreatic SOD activity in *R. philippinarum* increased significantly at 3 h, but was reduced at 12 and 24 h (*P* < 0.05); it increased again after reimmersion (Figure 4A). Under aerial exposure at high temperature, the hepatopancreatic SOD activity in *R. philippinarum* was significantly elevated at 3 and 6 h, and then increased significantly at 6 and 12 h after reimmersion (*P* < 0.05) (Figure 4B).

### Effects of Aerial Exposure on PHD Activity in Manila Clam

**FIGURE 5:**
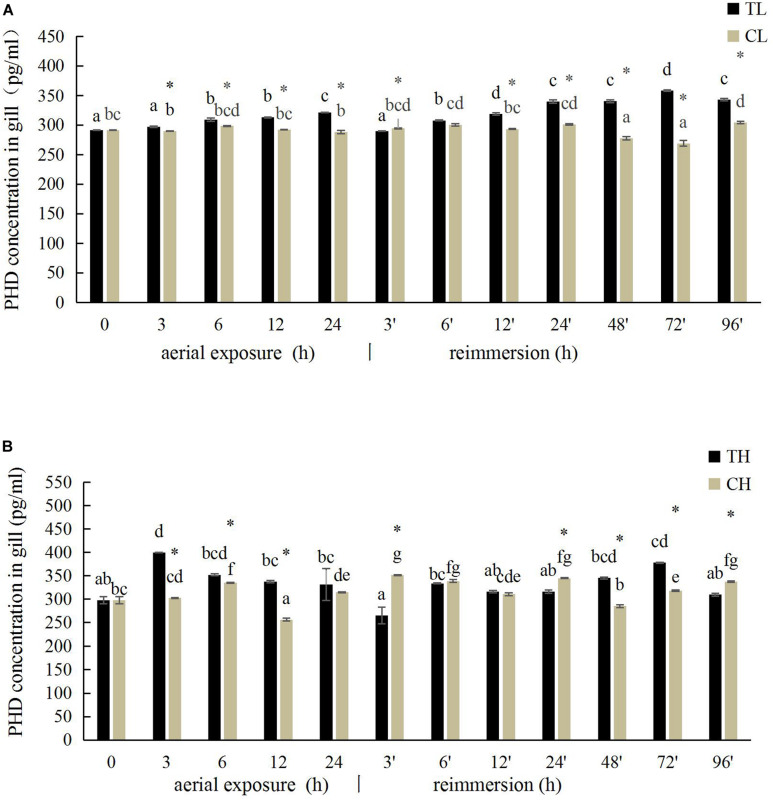
Effects of aerial exposure on PHD in gill of *R. philippinarum* under low temperature **(A)** and high temperature **(B)**.

**FIGURE 6:**
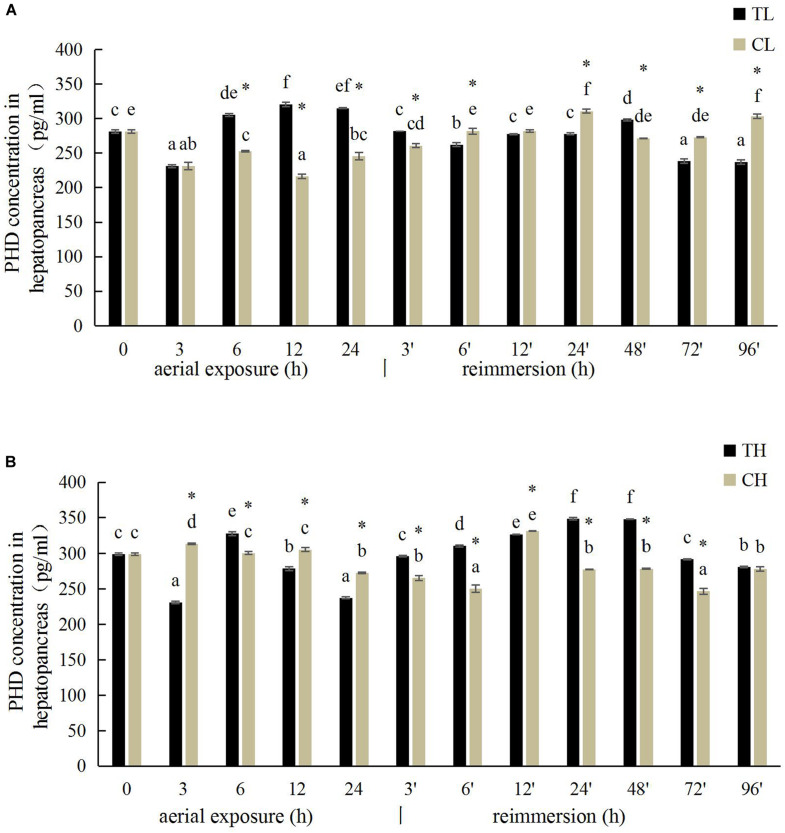
Effects of aerial exposure on PHD in hepatopancreas of *R. philippinarum* under low temperature **(A)** and high temperature **(B)**.

The PHD activity in the gill of *R. philippinarum* under aerial exposure at low temperature is shown in Figure 5A. It increased significantly at 6, 12, and 24 h under aerial exposure stress (*P* < 0.05), and when the calm was reimmersed, it first decreased and then increased (Figure 5A). In the hepatopancreas, the change trend of PHD activity was similar to the PHD activity in the gill (Figure 6A) Under aerial exposure at high temperature, the hepatopancreatic PHD activity was significantly reduced at 3 h, increased at 6 h (Figure 6B), whereas that in the *R. philippinarum* gill decreased at 12 and 24 h (Figure 5B). The PHD activity increased significantly from 3 to 48 h after reimmersion (*P* < 0.05).

### Mortality and Tissue Distributions of α-Amylase, SOD, and RpCTL

The mortality rates of the clams under aerial exposure for 24 h at low and high temperatures and within 120 h of reimmersion were calculated (Table 1). As shown in Table 1, there were no dead individuals within the first 24 h of aerial exposure at high temperature (28°C) and low temperature (4°C). The mortality rate was 1.11% after 12 h of reimmersion, and peaked (46.59%) at 48 h after reimmersion in high temperature group. After 48 h, the mortality rate began to decrease, and after 120 h, the mortality rate was 0. In contrast, there were no dead individuals after low-temperature aerial exposure (Table 1).

**TABLE 1:** Mortality of clams under aerial exposure at low and high temperatures for 24 h and after reoxygenation for 120 h.

Aerial exposure and reoxygenation time (h)	Mortality at 4°C (%)	28°C mortality (%)
**Aerial exposure time**		
0	0	0
3	0	0
6	0	0
12	0	0
24	0	0
**Reoxygenation time**		
3	0	0
6	0	0
12	0	1.11 ± 1.92
24	0	1.12 ± 1.94
48	0	46.59 ± 16.11
72	0	40.42 ± 13.29
96	0	14.28 ± 6.18
120	0	0

**FIGURE 7:**
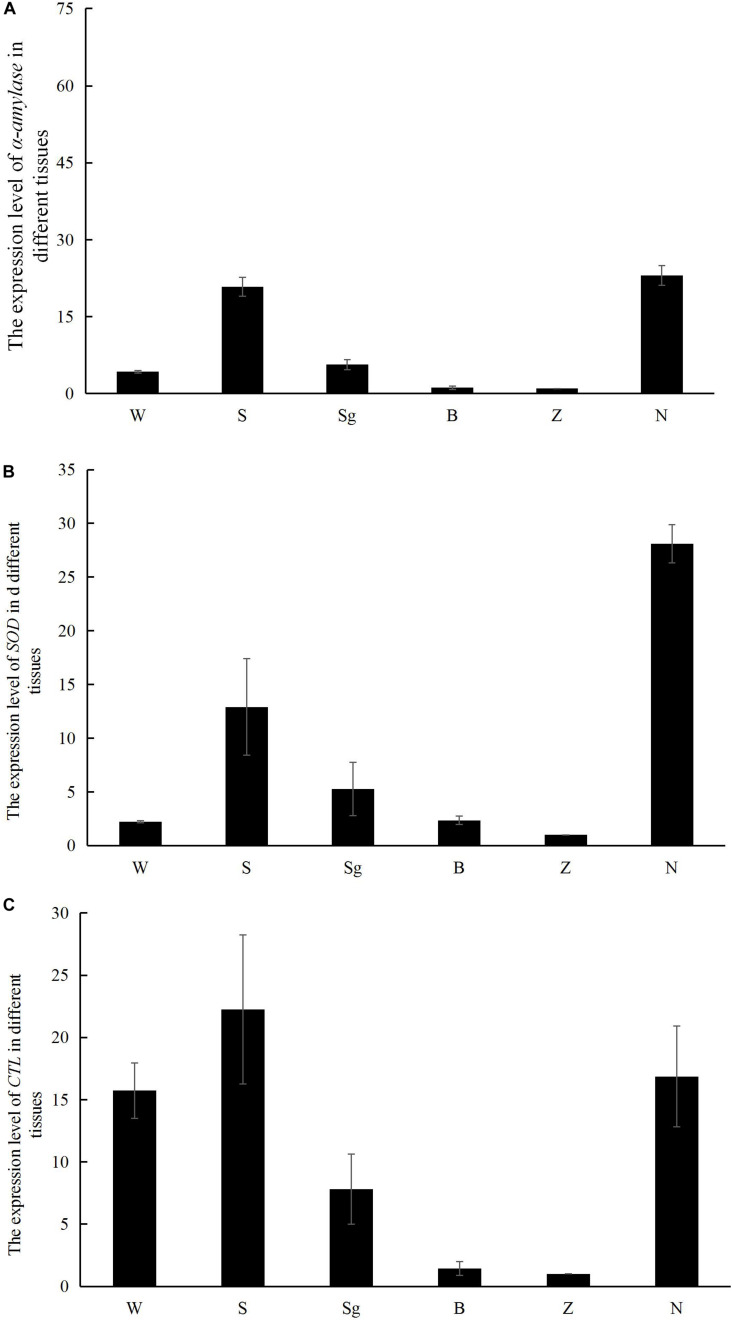
The mRNA expression of *α-amylase* **(A)**, *SOD* **(B)**, and *RpCTL* **(C)** in different tissues of Manila clam, including mantle (W), gill (S), siphon (sg), adductor muscle (B), foot (Z), and hepatopancreas (N).

The α-amylase and SOD transcripts were predominantly expressed in the hepatopancreas and gills, whereas RpCTL transcripts were highly expressed in the mantle, hepatopancreas, and gill (Figure 7).

### Expression Profiles of α-Amylase, SOD, and RpCTL mRNAs After Aerial Exposure and Reimmersion

**FIGURE 8:**
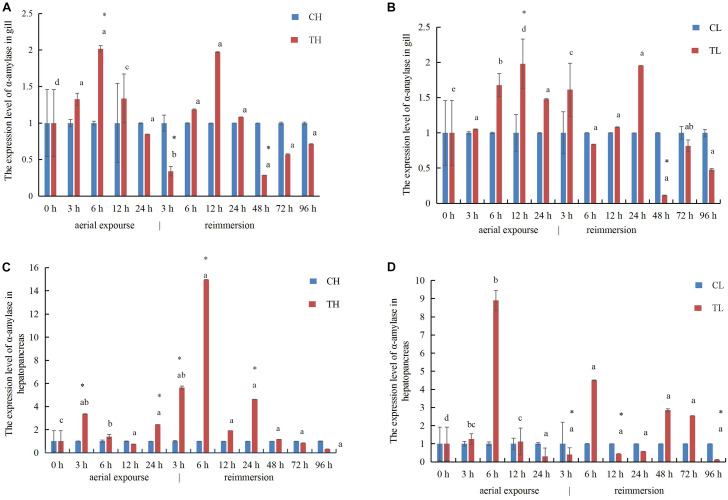
The expression analysis of α-amylase in the gill of *R. philippinarum* under aerial exposure and reimmersion at high **(A)** and low temperature **(B)**. The expression analysis of α-amylase in the hepatopancreas of *R. philippinarum* under aerial exposure and reimmersion at high **(C)** and low temperature **(D)**.

In the gills, the relative expression of α-amylase mRNA increased first and reached its highest level at 6 and 12 h under aerial exposure at high temperature and low temperature, respectively (Figures 8A,B), whereas α-amylase mRNA decreased at 3 h after reimmersion after aerial exposure at high temperature (Figure 8A). It then decreased at 48 h after reimmersion after both the high and low temperature treatments (Figures 8A,B). In the hepatopancreas, the relative expression of α-amylase mRNA increased at 3 and 6 h under aerial exposure at the high and low temperature, respectively (Figures 8C,D). The expression of α-amylase mRNA increased at 3 and 6 h and at 6 h after reimmersion following aerial exposure at both high and low temperature, respectively (Figures 8C,D).

**FIGURE 9:**
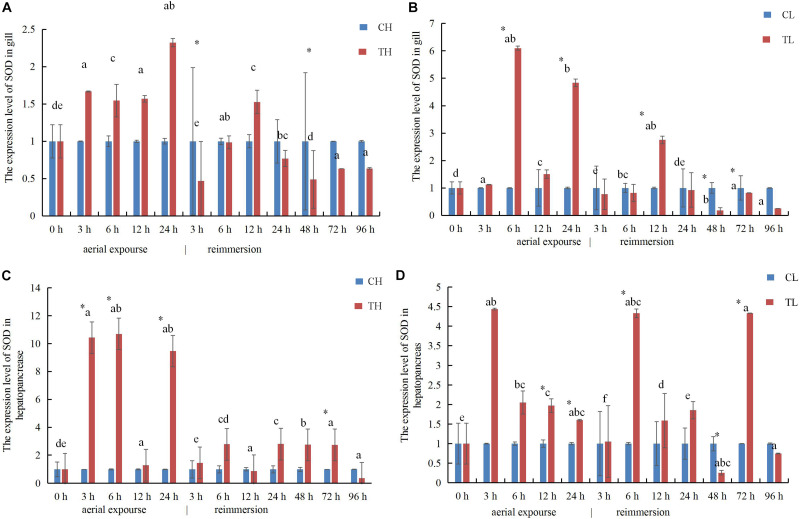
The expression analysis of *SOD* in the gill of *R. philippinarum* under aerial exposure and reimmersion at high **(A)** and low temperature **(B)**. The expression analysis of *SOD* in the hepatopancreas of *R. philippinarum* under aerial exposure and reimmersion at high **(C)** and low temperature **(D)**.

The expression of *SOD* mRNA was higher in the experimental group than in the control group at low temperature (Figures 9A,B). The expression of *SOD* mRNA in the gill increased first between 3 and 24 h under aerial exposure at high temperature, and then decreased significantly at 3 h after reimmersion (Figure 9A). The relative expression of *SOD* mRNA decreased in the gill at high temperature and increased 6 h again after reimmersion (Figure 9A). Under low-temperature aerial exposure, the expression of *SOD* mRNA in the gill increased significantly at 6 h, and again at 12 h after reimmersion (Figure 9B). The hepatopancreatic expression of SOD mRNA increased significantly at 3 h under aerial exposure at both low and high temperature (Figures 9C,D). The hepatopancreatic expression of *SOD* mRNA was significantly higher at 3, 6, and 24 h under aerial exposure at high temperature than in the control group, whereas in the low temperature group, it increased significantly at 6 h and fluctuated strikingly after reimmersion (*P* < 0.05).

**FIGURE 10:**
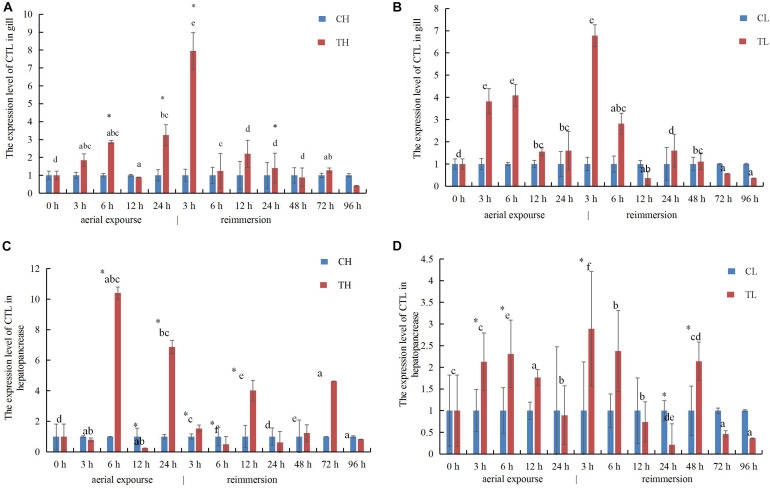
The expression analysis of *RpCTL* in the gill of *R. philippinarum* under aerial exposure and reimmersion at high **(A)** and low temperature **(B)**. The expression analysis of *RpCTL* in the hepatopancreas of *R. philippinarum* under aerial exposure and reimmersion at high **(C)** and low temperature **(D)**.

The relative expression of *RpCTL* mRNA in the gill increased at 3 and 6 h under aerial exposure at both low and high temperature, indicating that aerial exposure promoted the expression of the CTL gene. There was a further increase in *RpCTL* mRNA at 3 h after reimmersion (Figures 10A,B). *RpCTL* mRNA expression showed a similar trend and expression pattern in the hepatopancreas during aerial exposure (Figures 10C,D). The relative hepatopancreatic expression of *RpCTL* mRNA in the clams was downregulated after 3 h of aerial exposure at high temperature but upregulated at low temperature. The hepatopancreatic expression of *RpCTL* mRNA increased significantly after reimmersion for 3 h following aerial exposure at low temperature (*P* < 0.05) (Figures 10C,D).

## Discussion

During shellfish harvest and transportation, most shellfish will face the stress imposed by aerial exposure and temperature. Closing the shell is an important way for shellfish to protect themselves (Kawabe and Yokoyama, 2012). When the shell is closed during aerial exposure, the shellfish is stressed by the low oxygen availability, and the main energy supply mode in the shellfish body probably reverts to anaerobic respiration. The shellfish may then change its energy metabolism from anaerobic to aerobic during reimmersion. In shellfish, the respiration is positively correlated to temperature (Ali and Nakamura, 1999). Low temperature and humid environments can effectively reduce the aerial exposure stress response (Ali and Nakamura, 1999; Almeida and Bainy, 2006). It has been reported that temperature is the major factor affecting the water loss rate and tolerance of aerial exposure in *Corbicula fluminea* (Byrne et al., 1988). The tolerance time of *Helice tientsinensis* under aerial exposure was positively correlated with the relative environmental humidity (Sussarellu et al., 2010), and the survival rates of *R. philippinarum* and *C. fluminea* improved in low temperature and moist environments (Byrne et al., 1988; Ali and Nakamura, 1999). In this study, all the clams in the low temperature aerial exposure group survived after reimmersion, whereas a large number of clams in the high-temperature aerial-exposure group died at 48 h after reimmersion, indicating that low temperature can effectively reduce the stress of aerial exposure and improve the clam’s survival.

A mylase plays an important role in glycolysis, in which it hydrolyzes amylose to glucose and maltose (Yin et al., 2017). α-Amylase is a key enzyme in carbohydrate assimilation in mollusks and a possible rate-limiting enzyme in the metabolic pathway (Solidoro et al., 2000). In this study, α-amylase activity increased in the gills and hepatopancreas, but increased less markedly in the high-temperature group. Because the gill is the key tissue of respiration, α-amylase activity increases and decomposition produces more glucose under hypoxic conditions. After reimmersion, the α-amylase activity first fluctuated and then tended to become stable. This may be because reimmersion caused the clam to revert from anaerobic respiration to aerobic respiration. The α-amylase activity in the low and high temperature groups first increased and then tended to become stable, which may be attributable to the increased α-amylase activity induced by the digestion of food filtered from the water after reimmersion.

In this study, aerial exposure at high temperature led to reduced SOD activity after 3 h of reimmersion, which then increased and tended to become stable. This may be because oxidative damage to the body inhibited the activity of SOD, which then gradually increased after the body recovered from the stress response and self-adjusting. Our results showed that aerial exposure caused the SOD activity to improve early, to remove excess reactive oxygen species, and then decreased because of the adjustment of organism might be delayed, causing oxidative damage to the organism via oxidative stress and lipid peroxidation (Hiong, 2004). However, SOD activity was inhibited after reimmersion, and the enzyme activity increased in the early stage in both groups, possibly because any damage was repaired and self-regulation reestablished. The expression of the *SOD* gene after aerial exposure for 6 h was higher at the low temperature than at the high temperature, indicating that the expression of the *SOD* gene was more active under low-temperature conditions at this time, and the expression of the *SOD* gene after aerial exposure for 12 h was similar, indicating that its expression during aerial exposure for 6 h was enhanced.

Proline hydroxylase is a key regulator of the HIF pathway (Nguyen and Durán, 2016). In the HIF pathway, PHD controls the hydroxylation of key glycolytic enzymes, such as pyruvate kinase and pyruvate dehydrogenase. PHD may also control the rate of glycolysis in both HIF-dependent and non-HIF-dependent ways by regulating the activities of PKM2 and PDH2. The activity of PHD2 is inhibited by the tricarboxylic acid cycle (Barth et al., 2009). The inhibition of PHD stabilizes HIF and allows cells to adapt to hypoxia during periods of oxygen restriction (Serra-Perez et al., 2010). After reimmersion, HIF decreased and PHD expression was induced. The PHD activity in the hepatopancreas was inhibited after 3 h of low temperature aerial exposure, but became stable after 6, 12, and 24 h, possibly because PHD activity was inhibited to stabilize HIF in an adaptation to hypoxia. The PHD activity in the high temperature group was inhibited at 3 h, increased at 6 h, and decreased thereafter, perhaps because the PHD activity was inhibited to stabilize HIF as an adaptation to hypoxia. Its subsequent reduction may be attributable to the fact that the concentration of PHD induced by HIF decreased, and the adjustment was delayed.

C-type lectins are PRRs that play important roles in immune system of clams (Li et al., 2019). In this experiment, the expression of *RpCTL* increased to different levels during aerial exposure at low or high temperature. High temperature aerial exposure caused it to increase more dramatically than low temperature aerial exposure in gill and hepatopancreas tissues. These up- and down-regulated expression level of *RpCTL* were significantly greater in the high temperature group than in the low temperature group, indicating that high temperature aerial exposure had a greater effect on the expression of *RpCTL* than low temperature aerial exposure. Our results suggest that high temperature aerial exposure has a greater impact on the immune response of clams than low temperature aerial exposure, and that the expression of immune-related genes is significantly higher during high temperature exposure. This speculation is supported by the mortality observed during the whole experiment. All the individuals survived during low temperature aerial exposure for 24 h and reimmersion for 120 h. However, individuals died after reimmersion for 12 h following high-temperature aerial exposure, and the mortality rate was maximum at 48 h, at that time the expression level of *RpCTL* was down regulated.

## Conclusion

The changes in physiological indices of SOD, α-amylase and PHD during the process of resistance to air exposure were investigated by monitoring enzyme activities of *R. philippinarum* under the stress imposed by aerial exposure and reimmersion in water at different temperatures. At the same time, the expression level of *SOD*, *α-amylase* and *RpCTL* also were detected. We also monitored the different mortality rates during these processes under different conditions. These data indicate that transportation involving long-term aerial exposure should be undertaken in a low-temperature environment. This study provides new insights into the physiological and biochemical mechanisms underlying aerial exposure tolerance in *R. philippinarum* and extends our understanding of the physiological changes that occur in response to low oxygen availability, the fundamental adaptive physiology of this organism, and the molecular mechanisms operating during aerial exposure.

## Data Availability Statement

All datasets generated for this study are included in the article/Supplementary Material.

## Author Contributions

HN and XY conceived the study and revised the manuscript. KJ, ZZ, BG, and DL conducted the experiment. ZZ, BG, and DL analyzed the data. HN, KJ, and ZZ wrote the draft manuscript.

## Conflict of Interest

The authors declare that the research was conducted in the absence of any commercial or financial relationships that could be construed as a potential conflict of interest.

## Supplementary Material

FIGURE S1 The quality of the total RNA used in this study.

TABLE S1 The primers used in this study.

## References

[B1] Afonso V. Champy R. Mitrovic D. Collin P. Lomri A. (2007). Reactive oxygen species and superoxide dismutases: role in joint diseases. *Joint Bone Spine* 74 324–329. 10.1016/j.jbspin.2007.02.002 17590367

[B2] Ali F. Nakamura K. (1999). Effect of temperature and relative humidity on the tolerance of the Japanese clam, *Ruditapes philippinarum* (Adams & Reeve), to air exposure. *Aquac. Res.* 30 629–636.

[B3] Almeida E. A. Bainy A. C. D. (2006). Effects of aerial exposure on antioxidant defenses in the brown mussel *Perna perna*. *Braz. Arch. Biol. Technol.* 49 225–229.

[B4] Almeida E. A. Bainy A. C. D. Dafre A. L. Gomes O. F. Medeiros M. H. G. Di Mascio P. (2005). Oxidative stress in hepatopancreas and gill of the brown mussel (Perna perna) exposed to air and re-submersed. *J. Exp. Mar. Biol. Ecol.* 318 21–30.

[B5] Barth S. Edlich F. Berchner-Pfannschmidt U. Gneuss S. Jahreis G. Hasgall P. A. (2009). Hypoxia-inducible Factor Prolyl-4-hydroxylase PHD2 protein abundance depends on integral membrane anchoring of FKBP38. *J. Biol. Chem.* 284 23046–23058. 10.1074/jbc.M109.032631 19546213 PMC2755711

[B6] Byrne R. A. Gnaiger E. McMahon R. F. Dietz T. H. (1990). Behavioral and metabolic responses to emersion and subsequent reimmersion in the freshwater bivalve Corbicula fluminea. *Biol. Bull.* 178 251–259. 10.2307/1541826 29314943

[B7] Byrne R. A. Mcmahon R. F. Dietz T. H. (1988). Temperature and relative humidity effects on aerial exposure tolerance in the freshwater bivalve *Corbicula fluminea*. *Biol. Bull.* 175 253–260.

[B8] Ekblom R. Galindo J. (2011). Applications of next generation sequencing in molecular ecology of non-model organisms. *Heredity* 107 1–15. 10.1038/hdy.2010.152 21139633 PMC3186121

[B9] Giannetto A. Maisano M. Cappello T. Oliva S. Parrino V. Natalotto A. (2015). Hypoxia-inducible factor α and Hif-prolyl hydroxylase characterization and gene expression in short-time air-exposed *Mytilus galloprovincialis*. *Mar. Biotechnol.* 17 768–781. 10.1007/s10126-015-9655-7 26277612

[B10] Giannetto A. Maisano M. Cappello T. Oliva S. Parrino V. Natalotto A. (2017). Effects of oxygen availability on oxidative stress biomarkers in the mediterranean mussel *Mytilus galloprovincialis*. *Mar. Biotechnol.* 19 614–626. 10.1007/s10126-017-9780-6 29151140

[B11] Hiong K. C. (2004). Exposure to air, but not seawater, increases the glutamine content and the glutamine synthetase activity in the marsh clam *Polymesoda expansa*. *J. Exp. Biol.* 207 4605–4614. 10.1242/jeb.01334 15579556

[B12] Kawabe S. Yokoyama Y. (2012). Role of hypoxia-inducible factor alpha in response to hypoxia and heat shock in the pacific oyster *Crassostrea gigas*. *Mar. Biotechnol.* 14 106–119. 10.1007/s10126-011-9394-3 21748344

[B13] Li D. Nie H. Dong S. Huo Z. Yan X. (2019). Molecular cloning and expression analysis of C-type lectin (RpCTL) in manila clam *Ruditapes philippinarum* after lipopolysaccharide challenge. *Fish Shellf. Immunol.* 86 981–993. 10.1016/j.fsi.2018.12.033 30578844

[B14] Li Y. Lai S. Wang R. Zhao Y. Qin H. Jiang L. (2017). RNA-Seq analysis of the antioxidant status and immune response of *Portunus trituberculatus* following aerial exposure. *Mar. Biotechnol.* 19 89–101. 10.1007/s10126-017-9731-2 28138936

[B15] Livak K. J. Schmittgen T. D. (2001). Analysis of relative gene expression data using real-time quantitative PCR and the 2 (-delta delta c (t)) method. *Methods* 25 402–408. 10.1006/meth.2001.1262 11846609

[B16] Malagoli D. Casarini L. Sacchi S. Ottaviani E. (2007). Stress and immune response in the mussel *Mytilus galloprovincialis*. *Fish Shellf. Immunol.* 23 171–177. 10.1016/j.fsi.2006.10.004 17132471

[B17] McMahon R. F. (1988). Respiratory response to periodic emergence in intertidal molluscs. *Am. Zool.* 28 97–114.

[B18] Nguyen T. L. Durán R. V. (2016). Prolyl hydroxylase domain enzymes and their role in cell signaling and cancer metabolism. *Int. J. Biochem. Cell Biol.* 80 71–80. 10.1016/j.biocel.2016.09.026 27702652

[B19] Nie H. T. Jiang L. W. Chen P. Huo Z. M. Yang F. Yan X. W. (2017). High throughput sequencing of RNA transcriptomes in *Ruditapes philippinarum* identifies genes involved in osmotic stress response. *Sci. Rep.* 7:4953 10.1038/s41598-017-05397-8 PMC5504028 28694531

[B20] Nie H. T. Jiang L. W. Huo Z. M. Liu L. H. Yang F. Yan X. W. (2016). Transcriptomic responses to low temperature stress in the Manila clam, *Ruditapes philippinarum*. *Fish Shellf. Immunol.* 55 358–366. 10.1016/j.fsi.2016.06.008 27288255

[B21] Nogueira L. Mello D. F. Trevisan R. Garcia D. Da Silva Acosta D. (2017). Hypoxia effects on oxidative stress and immunocompetence biomarkers in the mussel *Perna perna* (Mytilidae. Bivalvia). *Mar. Environ. Res.* 126 109–115. 10.1016/j.marenvres.2017.02.009 28260615

[B22] Philipp E. E. R. Wessels W. Gruber H. Strahl J. Wagner A. E. Ernst I. M. A. (2012). Gene expression and physiological changes of different populations of the long-lived bivalve Arctica islandica under low oxygen conditions. *PLoS One* 7:e44621 10.1371/journal.pone.0044621 PMC3446923 23028566

[B23] Piontkivska H. Chung J. S. Ivanina A. V. Sokolov E. P. Techa S. Sokolova I. M. (2011). Molecular characterization and mRNA expression of two key enzymes of hypoxia-sensing pathways in eastern oysters *Crassostrea virginica* (Gmelin): hypoxia-inducible factor alpha (HIF-alpha) and HIF-prolyl hydroxylase (PHD). *Comp. Biochem. Physiol. Part D Genomics Proteom.* 6 103–114. 10.1016/j.cbd.2010.10.003 PMC3102143 21106446

[B24] Place S. P. Menge B. A. Hofmann G. E. (2012). Transcriptome profiles link environmental variation and physiological response of *Mytilus californianus* between Pacific tides. *Funct. Ecol.* 26 144–155. 10.1111/j.1365-2435.2011.01924.x 22563136 PMC3340984

[B25] Qian H. Zhao X. Zhao X. (2003). Regulation of α-amylase gene expression. *Northw. Agric. J.* 4 87–90.

[B26] Romero M. C. Ansaldo M. Lovrich G. A. (2007). Effect of aerial exposure on the antioxidant status in the subantarctic stone crab *Paralomis granulose* (Decapoda: Anomura). *Comp. Biochem. Physiol. C* 146 54–59. 10.1016/j.cbpc.2006.06.009 16890496

[B27] Sellos D. Moal J. Degremont L. Huvet A. Daniel J. Y. Nicoulaud S. (2003). Structure of amylase genes in populations of pacific cupped oyster (*Crassostrea gigas*): tissue expression and allelic polymorphism. *Mar. Biotechnol.* 5 360–372. 10.1007/s10126-002-0089-7 14719164

[B28] Serra-Perez A. Planas A. M. Núñez-O’Mara A. Berra E. García-Villoria J. Ribes A. (2010). Extended Ischemia prevents HIF1α degradation at reoxygenation by imparing prolyl-hydroxylation: role of Krebs cycle metabolites. *J. Biol. Chem.* 285 18217–18224. 10.1074/jbc.M110.101048 20368331 PMC2881746

[B29] Solidoro C. Pastres R. Melaku Canu D. Pellizzato M. Rossi R. (2000). Modelling the growth of tapes Philippinarum in northern adriatic lagoons. *Mar. Ecol. Prog. Ser.* 199 137–148. 10.3354/meps199137

[B30] Sussarellu R. Fabioux C. Le Moullac G. Fleury E. Moraga D. (2010). Transcriptomic response of the Pacific oyster *Crassostrea gigas* to hypoxia. *Mar. Genomics* 3 133–143. 10.1016/j.margen.2010.08.005 21798207

[B31] Wang X. (2010). *Survival Time And The Changing Cule Of Microorganisms And Physicochemical Indexes In Ruditapes Philippinarums During Keeping Alive Period.* Zhanjiang: Guangdong Ocean University.

[B32] Widdows J. Bayne B. L. Livingstone D. R. Newell R. I. E. Donkin P. (1979). Physiological and biochemical responses of bivalve molluscs to exposure to air. *Comp. Biochem. Physiol. A* 62 301–308. 10.1016/0300-9629(79)90060-4

[B33] Widdows J. Shick J. M. (1985). Physiological responses of *Mytilus edulis* and *Cardium edule* to aerial exposure. *Mar. Biol.* 85 217–232. 10.1007/BF00393242

[B34] Woo S. Jeon H. Y. Kim S. R. Yum S. (2011). Differentially displayed genes with oxygen depletion stress and transcriptional responses in the marine mussel, *Mytilus galloprovincialis*. *Comp. Biochem. Physiol. Part D Genom. Proteom.* 6 348–356. 10.1016/j.cbd.2011.07.003 21849267

[B35] Yin X. Chen P. Chen H. Jin W. Yan X. (2017). Physiological performance of the intertidal Manila clam (*Ruditapes philippinarum*) to long-term daily rhythms of air exposure. *Sci. Rep.* 27:41648 10.1038/srep41648 PMC5269718 28128354

[B36] Zhang G. F. Yan X. W. (2006). Development of new three-phase culture methods for Manila clam, *Ruditapes philippinarum*, farming in northern China. *Aquaculture* 258 452–461. 10.1016/j.aquaculture.2006.04.046

[B37] Zhang G. F. Yan X. W. (2010). *Clam Aquaculture.* Beijing: Science Press.

[B38] Zwaan A. (1977). Anaerobic energy metabolism in bivalve molluscs. *Oceanogr. Mar. Biol. A Rev.* 15 103–187. 10.1016/0305-0491(76)90247-9

