# Supplementary figures and images for: Modulated Expression and Activities of Ruditapes philippinarum Enzymes After Oxidative Stress Induced by Aerial Exposure and Reimmersion
Source: Front Physiol. 2020 May 25;11:500. doi: 10.3389/fphys.2020.00500 (PMC7261918; doi:10.3389/fphys.2020.00500)

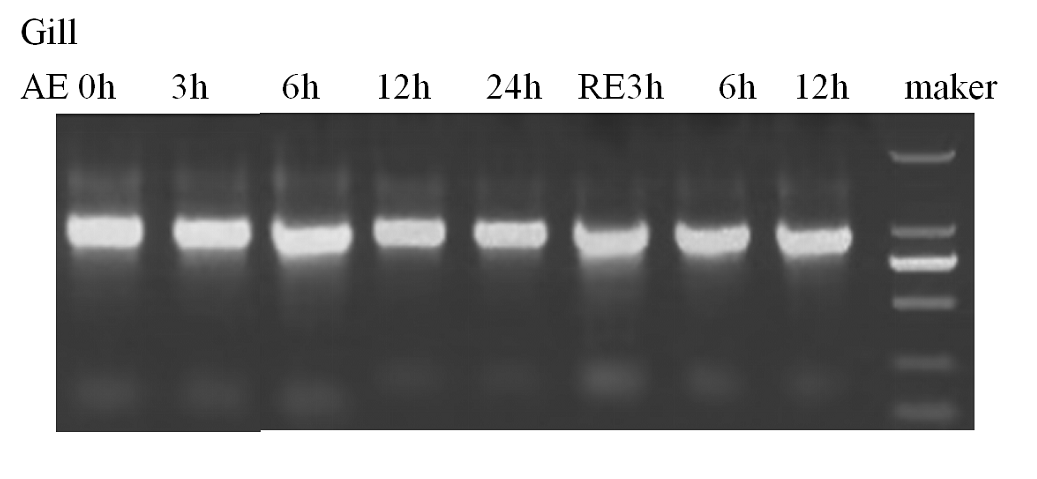

Supplement: FIGURE S1 — The quality of the total RNA used in this study. [file Image_1.tif]
