# Supplementary material for: Modulated Expression and Activities of Ruditapes philippinarum Enzymes After Oxidative Stress Induced by Aerial Exposure and Reimmersion
Source: Front Physiol. 2020 May 25;11:500. doi: 10.3389/fphys.2020.00500 (PMC7261918; doi:10.3389/fphys.2020.00500)
Supplement: TABLE S1 — The primers used in this study. [file Data_Sheet_1.pdf]

Table S1 The primers used in this study.

Primer	Sequence 5’-3’
β-actin-F	CTCCCTTGAGAAGAGCTACGA
β-actin-R	GATACCAGCAGATTCCATACCC
α-amylase-F	ACTTGGATTTGGGAACCTTCAC
α-amylase-R	TCGTCGCTCCATATATAATCAAAG
SOD-F	CGGGATCCGTGAAGGCTGTGGCTGTTC
SOD-R	CGGAATTCTTAACCTTGCAGACCAATAATGCC
C-Type-F	AGATTGATCAGCGGCGACCTTG
C-Type-R	CCTTCTGCGTGTCATCCAGTCC
